# Intrahemispheric dysfunction in primary motor cortex without corpus callosum: a transcranial magnetic stimulation study

**DOI:** 10.1186/1471-2377-6-21

**Published:** 2006-06-21

**Authors:** Shirley Fecteau, Maryse Lassonde, Hugo Théoret

**Affiliations:** 1 Centre de Recherche en Neuropsychologie et Cognition, Département de Psychologie, Université de Montréal and Hôpital Sainte-Justine, Montreal, Canada; 2 Center for Non-Invasive Brain Stimulation, Harvard Medical School, Beth Israel Deaconess Medical Center, Boston, USA; 3 Center for Non-Invasive Brain Stimulation, Harvard MedicalSchool, Beth Israel Deaconess Medical Center, 300 Brookline, Boston, MA 02215, USA; 4 Département de Psychologie, Université de Montréal, CP 6128, Succ. Centre-Ville, Montréal, QC, H3C 3J7, Canada

## Abstract

**Background:**

The two human cerebral hemispheres are continuously interacting, through excitatory and inhibitory influences and one critical structure subserving this interhemispheric balance is the corpus callosum. Interhemispheric neurophysiological abnormalities and intrahemispheric behavioral impairments have been reported in individuals lacking the corpus callosum. The aim of this study was to examine intrahemispheric neurophysiological function in primary motor cortex devoid of callosal projections.

**Methods:**

Intracortical excitatory and inhibitory systems were tested in three individuals with complete agenesis of the corpus callosum and sixteen healthy individuals. These systems were assessed using transcranial magnetic stimulation (TMS) protocols: motor threshold at rest, paired-pulse curve, and cortical silent period.

**Results:**

TMS revealed no difference between the patient and control groups on the motor threshold measure, as well as intracortical facilitation and intracortical inhibition systems as tested by paired stimulation. However, intrahemispheric inhibitory function was found to be abnormal in participants without callosal projections, as the cortical silent period duration was significantly increased in the patient group.

**Conclusion:**

These data suggest that in addition to previously reported impaired interhemispheric function, patients lacking the entire corpus callosum also display abnormal intrahemispheric excitability of the primary motor cortex.

## Background

The two human cerebral hemispheres are continuously interacting through excitatory and inhibitory influences and brain activity depends on this interhemispheric balance (see [1] for a review; [2,3]). One of the most efficient interhemispheric pathways and largest interhemispheric commissure at the cortical level is the corpus callosum (CC; [4,5]). Although anatomically the primary motor cortex (M1) is almost devoid of homotopic callosal connections, especially with respect to the distal representation of the hand and foot areas [6,7], physiologically potent interhemispheric interactions have been reported between M1s [8]. This interhemispheric communication presumably reflects active interaction mediated by fibers arising in motor association cortices [6,9]. Interhemispheric interactions between M1s have been widely studied in normal individuals and they appear to be composed of both inhibitory and facilitatory influences, with the inhibition between M1 hand representations being seemingly more prominent than facilitation [10,11]. Interhemispheric function has also been explored in individuals with agenesis of the CC. Meyer and collaborators reported that individuals with agenesis of the anterior trunk of the CC (subserving cortical motor areas) displayed absent or delayed transcallosal inhibition whereas this function was normal when the anterior trunk of the CC was spared [12].

Although numerous intrahemispheric inhibitory and excitatory phenomena have been studied in healthy individuals, the impact of total agenesis of the CC on intrahemispheric cortical function has not yet been specifically explored. This bears significant importance, as behavioral deficits in individuals with congenital absence of the CC are not limited to interhemispheric processing but also appear to be common place when intrahemispheric function is probed. Acallosal patients display intrahemispheric impairments in visual [13-17], visuomotor [16], and tactile processing [18]. Overall, it has been hypothesized that the absence of a CC may result in decreased neural activity within each hemisphere [17]. To assess intracortical excitability within each hemisphere independently, we used TMS [19-21] to probe facilitatory and inhibitory mechanisms occurring in M1. This goal prompted us to select a homogeneous group of unmedicated patients with *complete *agenesis of the CC. This patient feature allows the study of intracortical excitability in a motor cortex completely lacking callosal influences from the opposite hemisphere. The following measures were studied: resting motor threshold (MT), long-interval intracortical facilitation (ICF), short-interval intracortical inhibition (ICI), and cortical silent period (SP). We report deficits of intracortical function in acallosal patients.

## Methods

### Participants

Participants were naive to the purpose of the study. The study was approved by theethical committee from University of Montreal and all participants provided written informed consent prior to testing and were screened for contraindications to TMS [22]. None of the participants was under medication that could have an influence on central nervous system excitability [23].

#### Acallosal participants

Three individuals with complete agenesis of the CC took part in the study.

**Figure 1:**
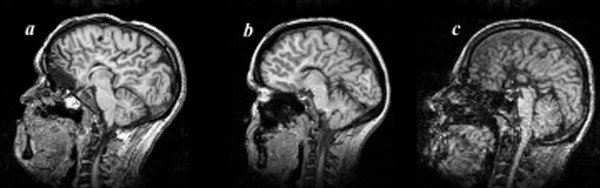
MRI images of patients in the sagittal plane. MRI image showing complete agenesis of the corpus callosum and the preserved anterior commissure of (A) patient M.G. and (B) patient S.G., as well as complete agenesis of both the corpus callosum and the anterior commissure of (C) patient S.Pe.

M.G. is a 36 year-old left-handed man with an IQ of 77. A complete agenesis of the CC with sparing of the anterior commissure has been revealed by MRI examination (Figure 1A). He is youngest of a family composed of other acallosal children. He experienced respiratory problems at birth and he was seen by a neurologist at 5 years old because of prolonged enuresis, poor motor coordination and retarded language acquisition. At age 8, the diagnosis of the agenesis of the corpus callosum was confirmed. He has finished school and he is currently unemployed (see also [24] for details).

S.G. is the sister of M.G. She is a 45 year-old right-handed woman with an IQ of 84 (see [25] for more details on her history). She also has a complete agenesis of the CC as revealed by CT and MRI scans, with preservation of the anterior commissure (Figure 1B). She was regarded as being asymptomatic except for a slow acquisition of walking, a symptom frequently associated with callosal agenesis. Her callosal agenesis was detected when she agreed to take part into a neuroradiological investigation of her family because of the presence of callosal agenesis in two of her siblings. She is presently working as an auxiliary nurse.

S.Pe., a 40 year-old right-handed man with an IQ of 107 (see [25] for his complete history), has a total agenesis of both the CC and the anterior commissure (Figure 1C). At the age of 18 months, a neonatal basal transpalatal encephalocele was surgically removed through a small bi-frontal craniotomy, which caused a discrete bilateral prefrontal atrophy. He is currently employed as an assistant manager in a drugstore.

#### Control participants

Sixteen healthy adults (three women; fourteen right-handed; mean age 33.8 ± 10.2) also took part in this study.

### Transcranial magnetic stimulation and recording technique

Participants sat comfortably during all TMS procedures and recording of motor-evoked potentials (MEP). TMS was performed with an 80-mm figure-of-eight coil and a Magpro X100 magnetic stimulator (Medtronic, Minneapolis, USA). The current wave form was biphasic and the orientation of the stimulation coil was 45° from the midline with the handle pointing backwards. Electrodes were placed over the contralateral first dorsal interosseus (FDI) muscle and a circular ground electrode was placed over the participants' wrist. The electromyographic signal was amplified using a Biopac MP150 system (Biopac Systems, Goleta, USA), filtered with a band pass of 20–1000 Hz and digitized at a sampling rate of 2000 Hz. Data were stored on a computer for off-line analysis. MEPs were recorded using Acqknowledge software (Biopac Systems, Goleta, USA). The following parameters were measured: MT at rest, responses to paired TMS pulses, and duration of the electromyographic silent period.

#### Motor threshold

The threshold for evoking MEPs in the contralateral FDI was first determined. MT was calculated as the minimal TMS intensity inducing MEPs greater than 50 μV in more than five out of ten trials in the FDI. For each participant, the stimulation point for eliciting maximal hand motor responses was determined individually (and for each hemisphere). This region was then stimulated during the entire experimental session. A tight-fitting lycra swimming cap was placed on the participant's head to mark the position of the TMS coil and to make sure that the same region was stimulated during the whole experiment.

#### Paired-pulse TMS

Subjects then participated in the paired-pulse experiment. We selected short interstimulus intervals (ISIs) of 1 and 2 ms to test intracortical inhibition and long ISIs of 9 and 12 ms to study intracortical facilitation according to the method of Kujirai et al. [26]. The conditioning stimulus was set at 80% of resting MT and the test stimulus at 120% of MT, which was adjusted to reproducibly induce MEPs of peak-to-peak amplitude of approximately 0.5 mV. We also included a test stimulus at 120% of MT that was used as baseline. Ten MEPs were recorded for each interval and for test stimulus alone. Thus, a total of 50 MEPs was collected for each hemisphere, with an intertrial interval set between 8 and 10 seconds. ISI and hemisphere order were pseudo-randomly varied across participants.

#### Silent period

Lastly, subjects participated in the SP experiment. The SP is a period of suppression of voluntary muscle contraction following a suprathreshold TMS pulse over the motor cortex [27]. The interpulse interval was set between 8 and 10 seconds. The intensity of TMS was set at 110%, 120% and 130% of the resting MT. Overall, 15 MEPs were collected for each hemisphere (5 MEPs for each intensity). Participants were asked to maintain an isometric voluntary muscle contraction of the contralateral FDI when single suprathreshold stimulation was delivered over the motor cortex. Muscle contraction was kept at 50% of maximum voluntary force using a force transducer providing an on-line digital readout so that participants could maintain their exerted strength at a constant level. Furthermore, each participant was instructed to relax quickly after the TMS pulse to preclude the 'instruction set' from modifying SP duration [28].

### Data analysis

Peak-to-peak amplitudes of the collected MEPs were measured. For MT, TMS-induced MEPs in the contralateral FDI were averaged and submitted to a two-way repeated measures analysis of variance (ANOVA) with *group *(controls, patients) and *hemisphere *(left, right) as factors. For the effects of paired stimulation, an ANOVA with *group, hemisphere *and *interstimulus interval *(1 ms, 2 ms, 9 ms, 12 ms) with repeated measures on the last two factors was performed. MEP amplitudes obtained through paired TMS were expressed as the percentage of the mean MEP amplitude over the test stimulus alone. To define the duration of SP, an experimenter blind to conditions calculated the time from the onset of stimulus delivery to the return of voluntary electromyographic activity based on visual analysis of the tracing. The electromyographic responses were averaged for each stimulation intensity. Data were then submitted to a repeated measures ANOVA with *group, hemisphere *and *intensity *(110%, 120%, 130% of resting MT) as factors. Within the sixteen healthy controls, eight participants were young adults and the other eight were age-matched with the acallosal patients (mean age (S.D.) of the controls = 42.1 ± 5.7 yrs and acallosal patients = 40.3 ± 4.5 yrs). We first compared the two control groups to explore age difference. Results revealed no significant difference between groups in the three measures (MT, paired TMS, and SP duration). Therefore, data from the two control groups (n = 16) were collapsed and then compared to those of the acallosal patients. Results with a P value ≤ 0.05 were considered significant for all statistical analyses.

## Results

None of the participants experienced any adverse effects during or after TMS. Only patient M.G. did not complete the SP experiment (at the intensity of 130% of the left hemisphere) because he felt tired.

### Motor threshold

In the control group, mean MT for the left and right hemispheres was 48.3% (SD = 6.3) and 50.0% (SD = 7.0), respectively. In the patient group, mean MT for the left hemisphere was 55.7% (SD = 9.5) and for the right hemisphere, 50.3% (SD = 11.7). Statistical analysis revealed no difference between groups (F = 0.98; n.s.) or hemispheres (F = 0.93; n.s.). Individual data for both patients and control participants are presented in Table 1[see Additional File 1].

### Paired-pulse

**Figure 2:**
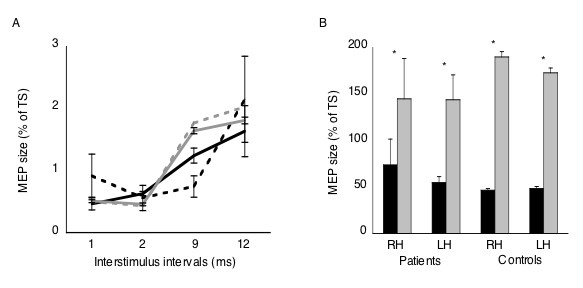
Intracortical inhibition and facilitation evoked by paired-pulse TMS. (A) The changes in MEP sizes of the contralateral FDI at the different interstimulus intervals. Black and gray lines show the percentage of MEP sizes for the patients and control groups, respectively. Continuous and dashed lines illustrate MEP amplitudes for the left and right hemisphere, respectively. (B) Size of MEPs for the short and long interstimulus intervals for each hemisphere and group. Black and gray columns show the percentage of MEP sizes for the short and long interstimulus intervals, respectively. RH: right hemisphere; LH: left hemisphere.

In both groups, the conditioning stimulus inhibited the test MEP at short ISIs (1 and 2 ms) whereas facilitation occurred at longer intervals (9 and 12 ms). MEP sizes were not significantly different between the two groups (Figure 2; F = 0.20; n.s.), or between the left and right hemispheres (F = 0.16; n.s.). As expected, there was a highly significant difference between ISIs (F = 23.53; P < 0.0001). Post-hoc tests with Bonferroni correction revealed that differences between the short and long ISIs were all highly significant (1 vs. 9 ms, 1 vs. 12 ms, 2 vs. 9 ms, 2 vs. 12 ms; all Ps < 0.05), whereas the two short and the two long ISIs were not significantly different from each other (1 vs. 2 ms, 9 vs. 12 ms; n.s.). Thus, we observed in both patient and control groups intracortical facilitation and intracortical inhibition of M1 in the left FDI evoked by paired stimulation of the right hemisphere, as well as of the right FDI by paired stimulation of the left hemisphere. Individual data are presented in Table 2 [see Additional File 2].

### Silent period

**Figure 3:**
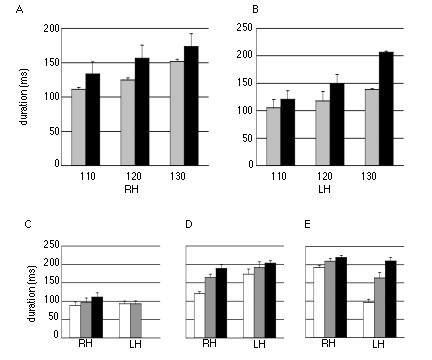
The effect of stimulus intensity on intracortical inhibition of primary motor cortex. TMS was performed with a focal suprathreshold stimulation over the motor cortex while electromyographic recordings were taken from the contralateral FDI muscle during voluntary tonic contraction. Silent period duration at different suprathreshold intensities for (A) the right and (B) left hemisphere for the control and patient groups in gray and black columns, respectively. White and gray columns show the silent period duration at the three intensities for the control and patient groups, respectively. (C) Silent period duration at the three intensities for the patient M.G., (D) S.G., and (E) S.Pe. Error bars indicate SEM. White (100%), gray (110%) and black (130%) columns show SP duration for the three different suprathreshold intensities. RH: right hemisphere; LH: left hemisphere.

**Figure 4:**
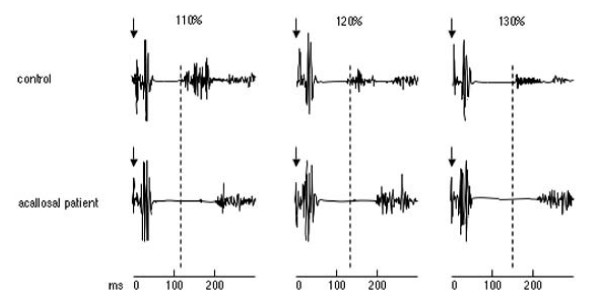
Examples of representative traces of the effect of stimulus intensity on intracortical inhibition in the left primary motor cortex of a control subject and the acallosal patient S.G.

Inhibitory function of the M1 as tested by the evoked cortical SP was found to be significantly different between groups. The patient group showed a greater SP duration than the control group for both hemispheres (*mean duration (s.e.m)of the left hemisphere*: patient = 153.0 ± 17.0 ms; control = 120.6 ± 2.3 ms; *mean duration (s.e.m) of the right hemisphere*: patient = 154.5 ± 17.0 ms; control = 129.3 ± 3.2 ms; ANOVA, F = 6.10; P = 0.02; Figure 3). Individual data for the patients are presented in Figure 3C, D, E. There was no significant effect of hemisphere (F = 0.08; n.s.). Overall, although there was a group difference, the duration of the SP increased proportionally with stimulation strength in both groups (F = 45.08; P < 0.0001). Representative examples of traces for two participants are shown in Figure 4.

## Discussion

The aim of this study was to explore the neurophysiology of the M1 devoid of callosal input. Using TMS, we report deficits of intracortical function in acallosal patients. That is, patients presented lengthened SP durations, whereas intracortical function as assessed by motor threshold and paired-pulse stimulation did not significantly differ between the patient and control groups. For the paired-pulse TMS, patients showed a lack of facilitation (although not significant) at 9 ms for the right hemisphere (see Figure 2). Although the exact mechanisms underlying these intracortical functions still have to be fully determined, the observed abnormalities suggest specific impairments of GABA-b receptors in our patient population.

Prior work has suggested that there might be intracortical deficits in patients with abnormalities of the CC. Indeed, a lack of transcallosal inhibition has been observed in this population [12] and it has been shown that disrupted transcallosal inhibition after an ischemic nerve block of one hand [30] can be accompanied by changes in intracortical inhibition. Moreover, studies focusing on the influence of interhemispheric inhibition on cortex excitability after stroke reported a reduced intracortical inhibition in the unaffected hemisphere following a disruption of transcallosal inhibition, while the duration of postexcitatory inhibition did not differ [31,32]. Furthermore, our results are in line with the reported intrahemispheric behavioral deficits of acallosal patients reported by Lassonde and colleagues [16-18]. Different explanations have been proposed to account for these dysfunctions. First, Lassonde suggested that congenital absence of the CC may result in a lower level of cortical activation [17]. That is, some specific cellular changes reported in callosal agenesis [33] may affect the responsiveness of each hemisphere. Lassonde and coll. also suggested that a lack of reinforcement by callosal input of cortico-cortical connections would account for the intrahemispheric deficits in this population [18]. We show here that faulty intracortical inhibitory mechanisms may underlie the reported behavioral deficits.

Intracortical inhibitory systems were assessed through short ISI paired stimulation and cortical SP paradigms. Paired stimulation at the two selected ISIs (1 and 2 ms) revealed no significant difference between the patient and control groups. The period of inhibition at ISIs of 1–5 ms has been ascribed to GABA-a inhibitory interneurons in the motor cortex [34], presumably different from those underlying long ICF [35]. This function appears to be preserved in the population studied here. These data are in agreement with a single-case report showing normal intracortical inhibition in a patient with a lesion of the entire CC due to an ischemic infarct [36]. However, patients seem to show a lack of intracortical inhibition at 1 ms (see Figure 2), although difference between patients and controls did not reach statistical significance. The lack of inhibition at this interval raises the possibility that the conditioning stimulation intensity was too high. It is possible that the conditioning stimulation was over the active motor threshold which would activate excitatory circuits. In healthy individuals, 80% of the resting MT usually represents the active MT [26]. However, inhibition at 2 ms was observed in these patients. Future studies with acallosal patients should include measures of active MT to investigate short SICI. Moreover, future work needs to include several short ISIs, as it has been suggested that different inhibitory mechanisms operate at ISIs of 1 ms and 2–5 ms [37,38]. Processes at 1 ms were found to be largely unaffected by neurotransmitter or neuromodulator systems in those studies which differentiated ICI at 1 ms and ICI at 2–5 ms. Inhibition at ISIs of 2–5 ms appears to occur mainly at the cortical level, whereas at 1 ms, inhibition may be caused by relative refractoriness of neural elements in the cortex activated by the conditioning pulse and the resulting desynchronization of the corticospinal volley [37,39], limiting explanatory value.

Intracortical inhibition was also tested using the cortical SP. We found that, whereas in both groups SP duration lengthened with increasing stimulus intensity, participants with agenesis of the CC showed significantly longer SPs. This enhanced inhibition suggests the existence of either deficient intracortical inhibition systems or reduced intracortical facilitatory mechanisms. SP abnormalities in agenesis of the CC have been reported by Meyer and coll. [12]. Using subthreshold stimulation intensities, they showed that in order to observe a postexcitatory SP following stimulation, higher stimulus intensities were needed in the patients compared to controls. We extend these result by showing that duration of the SP is also modified when interhemispheric interactions have been absent since birth. Damage to different brain areas can also cause a SP prolongation. For instance, SP has been reported to be prolonged in patients with lesions of the premotor cortex, lesions of the parietal and temporal lobe, and lesions of the internal capsule and thalamus [40]. As it is known that agenesis of the corpus callosum may be associated with a variety of central nervous system abnormalities, it is possible that these abnormalities are also involved in SP prolongation.

The duration of the SP has been attributed, at least its latter part, to the activity of intracortical inhibitory systems of the primary motor cortex, whereas spinal inhibition contributes to its early part [41]. It has been suggested that the latter part is caused by long-lasting cortical inhibition mediated by GABA-b receptors as the GABA re-uptake inhibitor tiagabine lengthens the SP [42]. L-DOPA and dopamine agonists also appear to lengthen SP duration [43]. Thus, GABA receptors seem to be crucial for the determination of the duration of SP. However, it seems that the involved intracortical GABAergic interneurons in SP are different from those involved in intracortical inhibition seen with paired pulse TMS [44]. Our data support this conclusion, as only SP duration was affected in our patient population, presumably through GABA-b receptors, whereas short ICI was normal.

**Figure 5:**
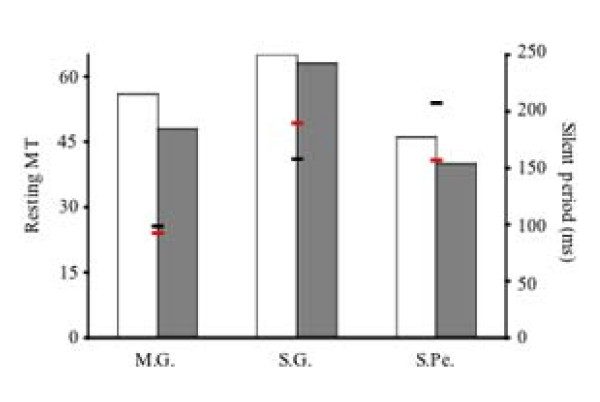
Resting motor threshold values and silent period duration for the patients. Primary *Y*-axis: averaged motor thresholds for each patient (% maximum stimulator output). White and gray columns show the motor threshold for the left and right hemisphere, respectively. Secondary *Y*-axis: silent period duration for each patient. Black lines show silent period duration in ms.

It is important to mention that SP duration may depend on a variety of factors in addition to potentially being a measure of GABA-b mediated cortical inhibition. We cannot entirely rule out the alternative possibility that abnormally lengthened SP duration observed in the patient group was related to the trend towards a higher resting MT, but this explanation seems unlikely. However, as shown in Figure 5, higher MTs observed in our patients do not seem to be related to SP duration. For example, a low motor threshold in patient S.Pe. is associated with a lengthened SP whereas in patient M.G. the opposite trend is observed. Moreover, SP abnormalities in agenesis of the CC have been reported by Meyer and coll. [12] using a different method. Using fixed stimulation intensities for patients and controls, they showed that in order to observe a postexcitatory SP following stimulation, higher stimulus intensities were needed in patients.

An alternative possibility is that abnormally lengthened SP duration observed in the patient group was related to the influence of motor set, motor instruction, or motor attention [28,45,46]. For example, patients with deficits in motor attention showed prolonged SP [45]. Thus, one could argue that the prolonged SP observed in our patients arises from a deficit in attentional processes. However, this appears unlikely as our participants understood the task and maintained a sustained voluntary contraction during the experiment as measured by the force transducer. Moreover, motor capability in these three patients was normal at the dynamometer. Additionally, to prevent the known influence of instructions given to subjects on SP duration [28], all participants were clearly instructed to quickly relax their index finger after each TMS pulse.

Cortical plasticity has often been reported in callosal agenesis [47]. For instance, our callosal agenesis individuals display normal performances on interhemispheric transfer tasks, including those requiring bimanual comparisons, a finding which is thought to reflect compensatory mechanisms. At a physiological level, the congenital loss of inhibitory callosal afferents may have been compensated for by increased local inhibition. Most likely, GABA would be mediating this increased inhibition, as it is thought to underlie the cortical aspects of the SP. In light of the suggested role of interhemispheric inhibition in fine motor control [48], the altered pattern of intracortical inhibition reported here may thus contribute to the relatively spared motor abilities of acallosal individuals. Whether the net effect of this abnormal physiological activity at the level of M1 is negatively or positively reflected in motor behavior, however, remains to be determined.

## Conclusion

In conclusion, we have presented evidence of impaired motor cortex inhibitory mechanisms in both cerebral hemispheres of individuals without a CC. Specifically, we have shown deficient intracortical inhibition presumably reflecting abnormal GABA-b mediated neurotransmission. Together with previous behavioral and neurophysiological data, these findings show that impairments are not restricted to interhemispheric interactions and that fundamental neurophysiological changes occur *within *each hemisphere when devoid of callosal projections. Future work needs to specifically address the question of GABA-b involvement in abnormal motor cortex inhibitory function and intrahemispheric behavioral impairments in acallosal individuals, possibly through pharmacological manipulations.

## Competing interests

The author(s) declare that they have no competing interests.

## Authors' contributions

All authors made substantial contributions to conception and design of the study. SF and HT carried out the TMS experiments. All authors were involved in statistical analysis and interpretation of data. All authors were involved in drafting the manuscript and have read and approved the final manuscript. HT supervised the study.

## Pre-publication history

The pre-publication history for this paper can be accessed here:



## Supplementary Material

Additional File 1 Motor threshold. Size of MEPs for the motor threshold for each participant and hemisphere. Click here for file

Additional File 2 Intracortical inhibition and facilitation evoked by paired-pulse TMS. Size of MEPs for the short (1 and 2 ms) and long interstimulus intervals (9 and 12 ms) for each participant and hemisphere. Click here for file
