# Supplementary material for: Intrahemispheric dysfunction in primary motor cortex without corpus callosum: a transcranial magnetic stimulation study
Source: BMC Neurol. 2006 Jun 21;6:21. doi: 10.1186/1471-2377-6-21 (PMC1513595; doi:10.1186/1471-2377-6-21)
Supplement: Additional File 1 — Motor threshold. Size of MEPs for the motor threshold for each participant and hemisphere. [file 1471-2377-6-21-S1.pdf]

Table 1

Participants	Motor threshold	
	Left hemisphere	Right hemisphere
M.G.	56	48
S.G.	65	63
S.Pe.	46	40
Control 1	39	41
Control 2	46	56
Control 3	55	50
Control 4	46	51
Control 5	47	53
Control 6	46	56
Control 7	36	38
Control 8	58	45
Control 9	45	53
Control 10	53	55
Control 11	46	41
Control 12	51	44
Control 13	54	55
Control 14	54	61
Control 15	48	50
Control 16	50	51
