# Supplementary material for: Intrahemispheric dysfunction in primary motor cortex without corpus callosum: a transcranial magnetic stimulation study
Source: BMC Neurol. 2006 Jun 21;6:21. doi: 10.1186/1471-2377-6-21 (PMC1513595; doi:10.1186/1471-2377-6-21)
Supplement: Additional File 2 — Intracortical inhibition and facilitation evoked by paired-pulse TMS. Size of MEPs for the short (1 and 2 ms) and long interstimulus intervals (9 and 12 ms) for each participant and hemisphere. [file 1471-2377-6-21-S2.pdf]

Table 2

Participants	Paired-pusle TMS							
	Left hemisphere				Right hemisphere			
	1 ms	2 ms	9 ms	12 ms	1 ms	2 ms	9 ms	12 ms
M.G.	0.39	0.70	1.53	2.88	0.25	0.18	0.87	0.88
S.G.	0.78	0.67	0.84	1.63	2.12	1.27	0.17	4.56
S.Pe.	0.22	0.52	1.36	0.40	0.39	0.22	1.19	1.02
Control 1	0.46	0.26	1.04	2.76	0.39	0.24	1.30	1.22
Control 2	0.04	0.05	0.93	0.32	0.57	0.66	2.72	6.13
Control 3	0.78	0.84	1.90	2.03	0.98	0.81	1.32	1.42
Control 4	0.52	0.41	1.57	2.53	0.17	0.22	0.89	0.93
Control 5	0.07	0.36	1.38	0.74	0.21	0.59	2.57	2.72
Control 6	0.54	0.64	1.20	1.18	0.89	0.64	1.85	1.94
Control 7	0.12	0.31	0.80	1.09	0.05	0.16	1.62	0.87
Control 8	0.12	0.05	1.30	1.12	0.12	0.08	1.52	1.35
Control 9	0.58	0.60	0.70	0.89	0.49	0.37	0.89	0.44
Control 10	0.70	0.12	2.26	2.27	0.42	0.30	1.33	1.17
Control 11	0.11	0.16	0.77	0.46	0.46	0.49	0.67	1.21
Control 12	0.35	0.23	0.61	0.85	0.50	0.59	1.58	2.17
Control 13	0.38	0.23	1.00	1.44	0.34	0.22	1.20	1.33
Control 14	0.49	0.46	1.71	2.84	0.61	0.26	2.22	2.03
Control 15	0.35	0.32	1.50	1.75	0.37	0.13	1.56	1.56
Control 16	0.29	0.36	1.37	1.07	0.45	0.50	3.68	5.19
